# Supplementary material for: The genome sequence of the Lapland Ringlet, Erebia embla (Thunberg, 1791) (Lepidoptera: Nymphalidae)
Source: Wellcome Open Res. 2026 Sep 4;11:588. [Version 1] doi: 10.12688/wellcomeopenres.27701.1 (PMC13631457; doi:10.12688/wellcomeopenres.27701.1)
Supplement: Peer review history

# Reviewer report: Reviewer response for version 1

**DOI:** 10.21956/wellcomeopenres.30492.r168640

**Published:** 2026-10-01

**Authors:** Savarimuthu Ignacimuthu, Sivasankaran Kuppusamy

**Affiliations:** 1 St Xavier's College, Palayamkottai, Tamil Nadu, India; 2 Loyola College Chennai, Tamil Nadu, India

The authors have generated the chromosome-level genome sequence of Lapland Ringlet, *Erebiaembla* (Thunberg, 1791). The genome assembly consists of two haplotypes. They assembled and annotated the first haplotype at the chromosome level, and the second at the scaffold level. The authors have used appropriate methods and software for high-molecular-weight DNA isolation, SMRTbell library preparation, assembly, and annotation. The comprehensive data will support phylogenomic analysis of Nymphalidae butterflies.

Comments on the manuscript

 The authors have identified a total of 15,659 protein-coding genes through genome assembly and have mentioned this number in the abstract of the manuscript. However, the information is not provided in Table 2. The authors may include the number of protein-coding genes in Table 2 for completeness.

 The authors have generated a total of 160.04 Gb of sequencing data using PacBio, Illumina, and RNA sequencing platforms. However, the authors haven’t provided information on the identification and annotation of non-coding genes and gene transcripts. Any reason?

 Overall, the research article is prepared well, and the manuscript meets the necessary scientific standards. It is suitable for indexing.

Are the datasets clearly presented in a usable and accessible format, and the assembly and annotation available in an appropriate subject-specific repository?

Yes

Are sufficient details of the sequencing and extraction, software used, and materials provided to allow replication by others?

Yes

Are the rationale for sequencing the genome and the species significance clearly described?

Yes

Are the protocols appropriate and is the work technically sound?

Yes

Reviewer Expertise:

Biotechnology, Molecular Biology

We confirm that we have read this submission and believe that we have an appropriate level of expertise to confirm that it is of an acceptable scientific standard.

# Reviewer report: Reviewer response for version 1

**Published:** 2026-09-04

**Authors:** Automated Benchmarking

**Affiliations:** 1 Wellcome Sanger Institute, Hinxton, UK

As this article is a Genome Note, Automated Benchmarking has been used to determine whether the reported assembly passes three defined checks established by the Earth BioGenome Project: the raw and assembled data are publicly available, the Earth BioGenome Project three-part metric is met, and the BUSCO score passes the stated threshold. For Erebia embla (ilEreEmbl2), all three checks passed. Data available: pass. ENA study PRJEB80074, raw-read accessions ERR13660089, ERR13670048, and ERR14986713, and assembly accessions GCA_964271835.1 and GCA_964271675.1 are public. Earth BioGenome Project three-part metric: pass. Haplotype 1 is reported as 6.C.Q65, meeting the 6.C.Q40 reference standard. BUSCO: pass. Haplotype 1, which is proposed as the reference assembly for this species, has 98.3% complete BUSCOs (97.8% single-copy and 0.5% duplicated). It meets the target of greater than 90% complete and single-copy BUSCOs. This decision uses values reported in the Genome Note and public accession records.

Reviewer Expertise:

NA

I confirm that I have read this submission and believe that I have an appropriate level of expertise to confirm that it is of an acceptable scientific standard.
